# Energy Consumption and Environment Performance Analysis of Induction-Healed Asphalt Pavement by Life Cycle Assessment (LCA)

**DOI:** 10.3390/ma14051244

**Published:** 2021-03-05

**Authors:** Qi Jiang, Fusong Wang, Quantao Liu, Jun Xie, Shaopeng Wu

**Affiliations:** 1 State Key Laboratory of Silicate Materials for Architectures, Wuhan University of Technology, Wuhan 430070, China; jiang7001@whut.edu.cn (Q.J.); wangfs@whut.edu.cn (F.W.); liuqt@whut.edu.cn (Q.L.); xiejun3970@whut.edu.cn (J.X.); 2 Department of Civil and Environmental Engineering, Norwegian University of Science and Technology, 7491 Trondheim, Norway

**Keywords:** energy consumption, greenhouse gas emissions, induced healing, LCA

## Abstract

In this paper, the sustainability of induced healing asphalt pavement is demonstrated by comparing the impact of asphalt pavement maintained by induced healing asphalt pavement technology and traditional maintenance methods (such as milling and overlaying). The functional unit selected is a 1-km lane with an analysis period of 20 years. The stages to be considered are material manufacturing, paving, maintenance, milling and demolition. Two case studies were analyzed to assess the impact of different technologies on the energy consumption and environmental performance of each maintenance alternative. By comparing the energy consumption and environmental emissions of the whole life cycle of pavement under the two technical conditions, the results show that the total energy consumption of traditional asphalt pavement is about 2.5 times that of induction-healed asphalt pavement, and the total greenhouse gas (GHG) emissions of the former are twice as much as that of the latter.

## 1. Introduction

In 2020, China’s fixed asset investment in highway transportation exceeded 2.2 trillion RMB [1]. By 2020, the total length of highways in China had reached 5.0125 million kilometers, an increase of 166,000 km over the previous year. The total length of roads maintained was 4.9531 million kilometers, accounting for 98.8% of the total highway length. One of the main reasons for the high amount of highway maintenance is that the current traffic presents the characteristics of large vehicle flow, large passenger volume and multi-channelization, which makes the asphalt pavement spall, crack, and creates potholes and other situations during the service period, and seriously affects the driving comfort of asphalt pavement [2,3]. In order to solve the problems caused by these situations, China mainly adopts the grouting joint, thin slurry sealing layer, micro surfacing and overlay maintenance measures [4]. However, it is difficult to diagnose the maintenance time of damaged pavement, so these pavement maintenance measures find it difficult to play a full role. Some of the pavement maintenance measures even cause traffic congestion, wastage of resources, high maintenance costs and environmental pollution due to the long maintenance time [5]. Therefore, it is urgent to study a new road maintenance technology in order to achieve the goal of fast and efficient road maintenance.

Electromagnetic induction (EMI) heating self-healing technology is a new efficient and fast maintenance measure for asphalt pavement. The concept of induction technology, originally developed by TU Delft, relies on conductive materials such as steel fibers, metal particles, and metal powders that can be inductively cured in asphalt mixtures [6]. The self-healing ability of asphalt concrete is highly dependent on temperature. When the temperature is too low, the penetration, diffusion and bonding of asphalt molecules between the fracture surfaces of asphalt concrete are weakened, leading to the difficulty of crack healing. However, with the increase in temperature, the infiltration, diffusion and bonding of asphalt molecules on the fracture surface is strengthened, which can significantly improve the healing rate of asphalt concrete. The self-healing technique of electromagnetic induction heating selectively heats the heat-induced materials such as steel fiber in asphalt mortar. The asphalt mortar containing steel fiber is heated up rapidly under the action of induction eddy current, which promotes the rapid healing of micro cracks, while the stone cannot be heated by induction heating. This local heating can not only ensure the healing of asphalt mortar cracks, but also avoid the damage to the pavement structure. Therefore, electromagnetic induction heating self-healing technology has a good application prospect [7].

In order to analyze the sustainability of induced healing techniques, life cycle assessments (LCA) were carried out according to ISO14040:2006 and 14044:2006 standards, which set out requirements and guidelines to be followed for proper analysis [8,9]. Life cycle assessment is a process to assess the environmental burden associated with a product, process or activity by identifying and quantifying the energy and materials used and wastes released to the environment, in order to assess the environmental impact of these energy and material uses and releases to the environment, and to identify and assess opportunities to affect environmental improvements. The assessment includes the entire life cycle of a product, process, or activity, including the extraction and processing of raw materials; manufacturing, transportation and distribution; use; reuse and maintenance; recycling and final disposal [10]. This methodology has previously been used to determine the environmental performance of pavements, principally comparing rigid and flexible layers [11,12], but has also investigated more innovative solutions, such as warm mixed asphalt or recycled materials [13,14,15]. However, there is little documentation of its use in the evaluation of induction-healing technology.

The purpose of this paper is to assess the energy consumption and greenhouse gas (GHG) emissions of different asphalt pavement maintenance technologies, to demonstrate the prospect of sustainable development of EMI heating self-healing technology. Since GHG emissions account for a major part of all air pollutants, and carbon dioxide is the main representative component of GHG, GHG are adopted as the main indicator of environmental impact in this paper [16]. Based on the test section project of self-repairing asphalt pavement, the life cycle of self-repairing asphalt pavement is divided into four stages: the material manufacturing stage, road construction and paving stage, road service maintenance stage and mixing mill recovery stage. According to the ISO 14040, the LCA method of shunt path analysis is used to assess the sustainability of self-repairing asphalt pavement by calculating the input of energy and resources and the output of air pollution in a separate process. 

A LCA is commonly divided into four steps: goal and scope, inventory analysis, impact assessment and interpretation of the results. The interpretation of the results can compare the environmental impact of materials and identify the strengths and weaknesses of the assessment process. This, in turn, can be used as part of the research process to improve and update technology and enhance competitiveness.

## 2. Goal and Scope

The goal of this LCA is to demonstrate the sustainability of induction-healed asphalt mixtures developed in the test section project of self-repairing asphalt pavement by comparing the environmental impacts that this new technology produces with that of traditional mixtures repaired by milling and covering technology. Since the induction-healing technology was developed only for the upper pavement layer, the analysis focused on that layer, leaving the rest of the pavement unchanged.

For the analysis, a steel fiber asphalt concrete self-healing test section of Jiehui Expressway in Guangdong Province of China is taken as an example; a highway with three lanes per direction and gradation of GAC-16C asphalt mixture was selected. The functional unit is defined as a 1-km lane with a width of 3.75 m and an upper layer thickness of 0.045 m. An analysis period of 20 years is assumed. 

**Figure 1:**
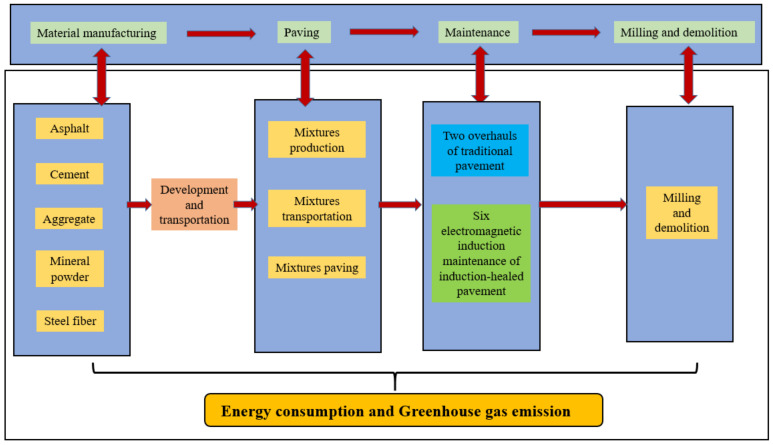
Life cycle assessment (LCA) boundaries.

The selection of the system boundaries is based on the stages defined in the standard EN 15804:2012 +A1:2003 [17,18]. However, in order to understand the integrity of the life cycle, the development and transportation of raw materials are considered as a separate module of the material manufacturing stage (Figure 1).

## 3. Life Cycle Inventory

This stage involves the creation of a consistent database by collecting and quantifying the inputs and outputs associated with the functional unit [12]. In this sense, data on resources consumed and emissions generated are compiled from different sources. Emissions from electricity production, fuel generation and combustion, and transportation (processes shared by all stages) were collected from the China Energy Statistical Yearbook (2018), Highway Life Cycle Energy Consumption Analysis and Energy Saving Strategy, and China’s localized LCA basic database [19,20,21,22]. In order to facilitate the calculation of energy consumption and GHG emissions at each stage of road construction in the life cycle, a list of major energy sources is listed, as shown in Table 1. The specific assumptions and data sources used to form the inventory for each stage are described next.

### 3.1. Material Manufacturing Stage

This stage includes resource consumption and GHG emissions from the development and transportation of the materials (asphalt, cement, coarse aggregate, mineral powder, fine aggregate, steel fiber). In addition to the raw materials of the traditional asphalt mixture, the key to the self-repairing asphalt pavement in this paper is the addition of steel fiber [16,23,24]. In the material manufacturing stage of raw materials, the production energy consumption and related emissions data of various materials are based on the existing database and relevant industrial environment and energy reports, while the transportation distance between the material manufacturer and the mixing station is recorded and converted to the calculated value according to the actual data of the project test section cases [25,26,27]. The upper layer production mix ratio of the test section is shown in Table 2, the production energy consumption and related emission data of various materials can be seen in Table 3, and the transportation distance is shown in Table 4.

Sources of raw materials used in the construction process of asphalt pavement are: asphalt produced in the Shell Styrene-butadiene-styrene (SBS) modified asphalt of Guangdong Yue Logistics Industrial Co., Ltd., Guangdong Province, China; aggregate with particle size of 3.5–22 mm is made from Shuangdong Furong Stone Farm; limestone fine aggregate and mineral powder with particle sizes of 0–2.36 mm are produced by self-processing; cement anti-spalling agent is produced by China Resources Cement Co., Ltd., Shenzhen, Guangdong Province, China; and conductive steel fiber is produced by Jiangsu Golden Torch Metal Products Co., Ltd., Jiangsu Province, China.

The production technology of self-repairing asphalt pavement is to add steel fiber with asphalt volume ratio of 6%, and increase asphalt dosage by 0.2% on the basis of the original mix ratio of ordinary asphalt concrete [28,29,30]. Considering the density difference between steel fiber and natural aggregate as well as binder, the proportion of added steel fiber and the later paving and rolling processes were optimized and adjusted accordingly.

According to the calculation of the existing production mix ratio in the project examples in the test section, the upper layer of 1 km traditional asphalt pavement needs 825.04 tons of coarse aggregate, 23.24 tons of cement, 290.51 tons of fine aggregate, 23.24 tons of mineral powder and 54.62 tons of asphalt. Accordingly, the 1 km self-repairing asphalt pavement needs 25.92 tons of steel fiber and 56.94 tons of asphalt.

### 3.2. Paving Stage

This stage includes three important processes: the preparation of asphalt mixture at the mixing station, the transportation of asphalt mixture to the paving site, and the paving and compaction of asphalt mixture. Compared to the traditional asphalt pavement construction, the self-repairing asphalt pavement is mixed with steel fiber, so the mixing time is 8 s longer than the conventional process requirements. Meanwhile, in order to ensure the compactness of the pavement, the workload of the paving and rolling process is increased.

The equipment selected for the mixing station is the forced intermittent asphalt mixture mixing equipment of Langfang Deji Machinery Technology Co., Ltd., Langfang, Hebei Province, China. The model is LB-4000 and the rated capacity is 320 t/h. In actual production, there is a big difference between the external mixing rate and the rated capacity [31,32]. Moreover, there will be quality loss of asphalt mixture in the actual construction process, so in the preparation process of the mixture, the actual quality of mixture is greater than the theoretical calculated quality, and the extra quality is controlled within 0.5%. Therefore, the actual energy consumption in the mixing stage is confirmed based on the parameters of rated capacity and the construction log records, as shown in Table 5.

In the transportation stage of the asphalt mixture, a dump truck is used to transport the mixture to the paving site. The single transport mass of the dump truck is 35 tons. Assuming that the mixing station is approximately 7 km from the paving site, according to the survey of the transport driver, the average consumption of diesel fuel per kilometer of is 2 L. Thus, 0.688 kg of diesel fuel is consumed of per ton of mixture transported.

In the paving and compacting stage of asphalt mixture, the main energy consumption is generated by the paver and rollers during the operation. Table 6 lists the main mechanical energy consumption in the paving stage of traditional asphalt pavement. The data in the table are from engineering log records and field mechanical driver surveys.

### 3.3. Maintenance Stage

Maintenance activities will depend on the mixture to be repaired as traditional asphalt mixtures will need to be replaced by rolling and covering techniques, and the induction-healed asphalt pavement will also be healed by induction-healing treatments [24,33,34]. Therefore, the impact of this stage is related to the two maintenance actions. The maintenance method of self-repairing asphalt pavement in this paper is to repair the pavement upper layer by electromagnetic induction heating by the maintenance vehicle, and the energy consumption is mainly generated by the electric energy consumed by the maintenance vehicle. Traditional asphalt pavement maintenance includes the impacts associated with the milling of the old asphalt layer, transportation to the recovery center, the production of materials, transportation to the roadworks and construction of new asphalt layer.

In order to determine the maintenance schedule (Table 7), it is necessary to stipulate the usual design life of traditional asphalt pavement. According to many previous experiences of construction enterprise, this can be estimated to be 15 years, while the design life of self-repairing asphalt pavement is 20 years [35,36].

During maintenance work, the power of the maintenance vehicle is 155 kW, the driving speed is 1.2 m/min, and the healing width is 5 m. Under this condition, the maintenance vehicle can raise the temperature of the upper layer of the road from the ambient temperature to the optimal healing temperature of 120 ℃ [37,38,39,40]. Therefore, the single maintenance of the 1 km asphalt pavement test section requires the maintenance vehicle to run twice and consumes 4305.56 kW·h of electric energy. The energy consumption of self-repairing asphalt pavement is 25,833.36 kW·h during the maintenance stage of its design life.

For the mill and overlay activities, according to the maintenance schedule of an overhaul in the seventh year and the twelfth year, respectively, the energy consumption at this stage mainly considers the materials and construction machinery involved in the overhaul. It is assumed that 50% reclaimed asphalt pavement (RAP) is used in the material manufacturing stage. Therefore, it is two processes of milling and overlaying for the overhaul of traditional asphalt pavement.

### 3.4. Milling and Demolition Stage

According to the survey of the test section project cases, when the milling machine runs at full load on the asphalt pavement, the milling machine consumes 0.11 L of diesel fuel for 1 ton of asphalt concrete. Assuming a distance of 40 km between the maintenance section and the mixing station, a 15-ton dump trucks is usually used to transport the used abrasive material and the newly mixed asphalt mixture. According to Budget Amount of Highway Engineering and Quota of Cost of Highway Construction Machinery Platform Shift [41,42], the energy consumption and GHG emissions generated in this stage are shown in Table 8.

## 4. Life Cycle Impact Assessment

Once the inventory was completed, the resources consumed and GHG emissions were converted into impacts through an energy conversion method, which calculated the energy by converting the standard coal coefficient to the standard coal multiple [12,25,32]. The environmental impact of each stage can be divided into two parts: energy consumption and CO_2_ equivalent emissions.

### 4.1. Energy Consumption Impact Assessment

**Figure 2:**
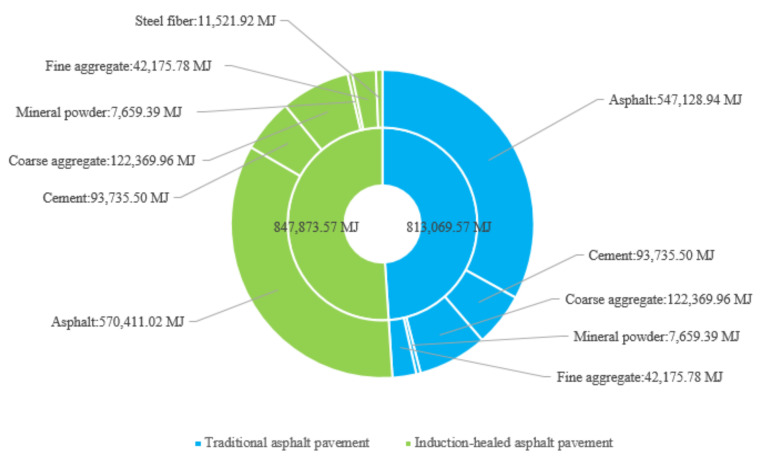
Energy consumption comparison between induction-healed pavement and traditional pavement in the material manufacturing stage.

**Figure 3:**
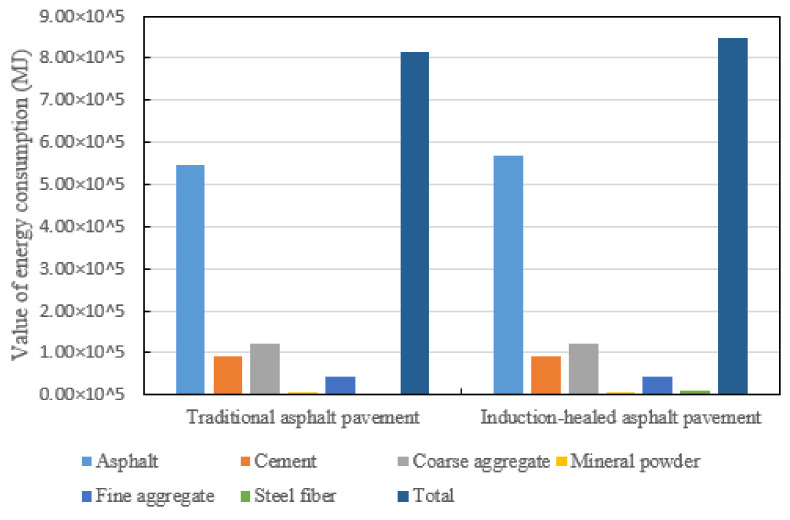
Fluctuation chart of energy consumption of the two asphalt pavements in the material manufacturing stage.

In the material manufacturing stage of raw materials, the energy consumption comparison between induction-healed pavement and traditional asphalt pavement is shown in Figure 2 and Figure 3. The pie chart shows the proportion of energy consumption in manufacturing and transportation for each material, and the bar chart shows the difference in the manufacturing and transportation energy consumption for each material. The energy consumption of induction-healed asphalt pavement at this stage is similar to that of traditional asphalt pavement, which are 51% and 49%, respectively. The production of asphalt is the most energy-intensive process in this stage, accounting for 67.28% and 67.29% in induction-healed asphalt pavement and conventional asphalt pavement, respectively.

**Figure 4:**
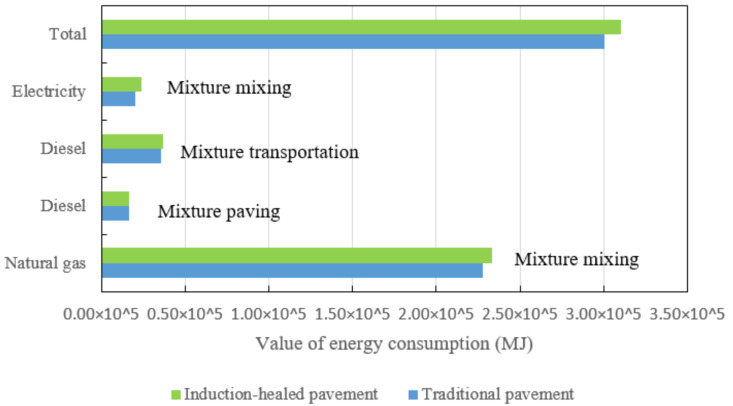
Energy consumption comparison of two asphalt pavement paving stages.

In the road paving stage, the energy consumption of induction-healed asphalt pavement was compared with that of traditional asphalt pavement, and the results are shown in Figure 4. The calculation of energy consumption in this stage is divided into three processes: mixing of the mixture, transportation of the mixture and paving of the mixture. As can be seen from the figure, the energy consumption of the induction-healed asphalt pavement at this stage is significantly higher than that of the traditional asphalt pavement, and the energy consumption of the pavement with the two technologies is the highest in the process of mixing the mixture. The reason for this result is that the quality and density of the mixture increase when the steel fiber is added to the former, resulting in an additional 8 s adjustment time in the mixing process and an increase in the amount of transportation time [29,34,39]. On the other hand, natural gas is the largest of the three types of energy consumed in this stage. The main reason is that only natural gas is used for energy supply during the mixing process of the asphalt mixture in the mixing station. Therefore, it can be inferred that the energy consumption of the asphalt mixture in the mixing process is the highest.

**Figure 5:**
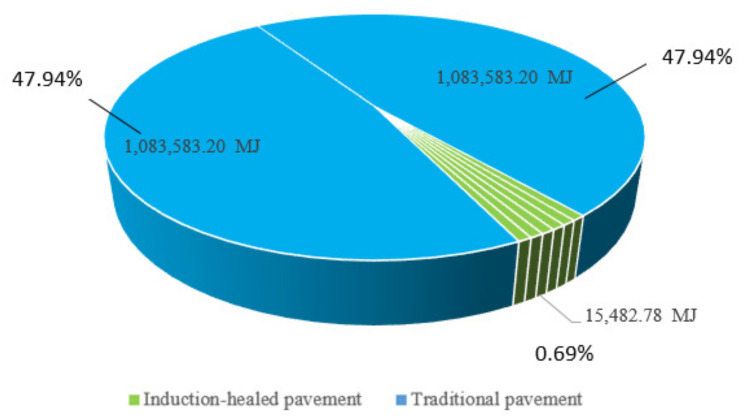
Energy consumption of two overhauls of traditional pavement and six electromagnetic induction (EMI) maintenance sessions of induction-healed pavement.

The biggest advantage of induction-healed pavement is that a simple and quick maintenance method can be used in the maintenance stage and less energy consumption can be generated. Using the maintenance vehicle to generate an electromagnetic field, the upper layer of the steel fiber of asphalt pavement due is heated to electromagnetic induction, so that the asphalt temperature increases to enhance mobility and repair pavement distress. As shown in Figure 5, the energy consumption of two overhauls of traditional asphalt pavement and six EMI maintenance sessions of induction-healed asphalt pavement are compared. According to the information shown in the figure, within the design life of two asphalt pavements, the energy consumption of a single EMI maintenance session of the induction-healed pavement is 15,482.78 MJ, accounting for 1.4% of the single overhaul of traditional asphalt pavement; the energy consumption of two overhauls of traditional pavement is 2,167,166.40 MJ, the total energy consumption of six EMI maintenance accounts for 4.3% of this figure.

**Figure 6:**
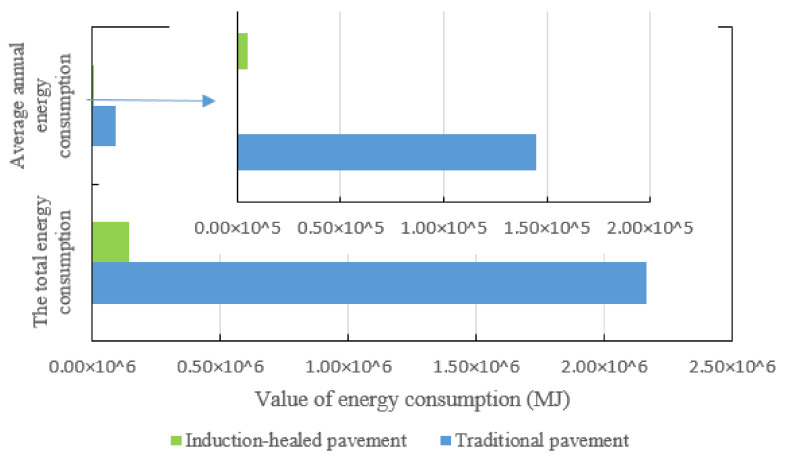
Energy consumption comparison of two asphalt pavements’ maintenance in their design life.

The design life of induction-healed asphalt pavement is 20 years, while the design life of traditional asphalt pavement is 15 years. According to the above pavement maintenance energy consumption, the average annual energy consumption of the two pavements can be obtained (Figure 6). The average annual energy consumption of induction-healed asphalt pavement is 3.2% of that of traditional pavement. It is obvious that the use of EMI maintenance technology can greatly reduce the energy consumption in the maintenance stage.

**Figure 7:**
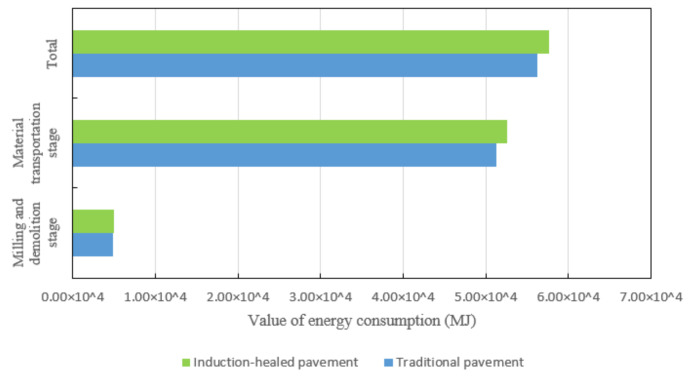
Energy consumption comparison of two asphalt pavements’ milling and demolition stages.

In the milling and demolition stage, the energy consumption is mainly generated in the transportation process of recycled materials. The addition of steel fibers to induction-healed asphalt pavement improves the quality and density of the asphalt mixture, resulting in greater energy consumption during milling and transportation (Figure 7).

**Figure 8:**
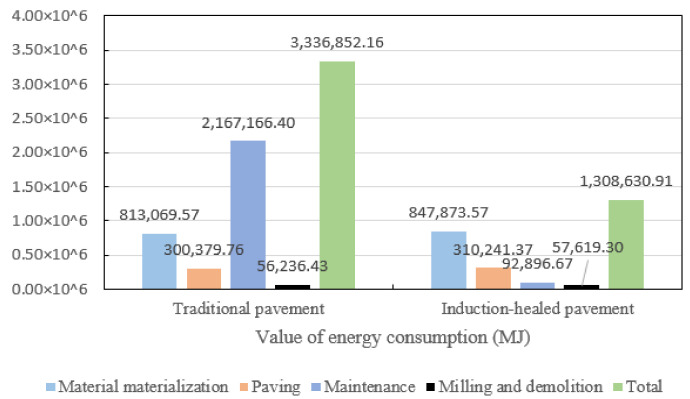
Energy consumption comparison of two asphalt pavements in their whole life cycle.

Based on the energy consumption in the four stages, the pavement life cycle energy consumption is shown in Figure 8. It can be seen from the figure that the energy consumption of the traditional pavement is about 2.55 times that of induction-healed pavement. Among the four stages, the energy consumption of induction-healed pavement is the largest in the material manufacturing stage, while that of traditional pavement is the largest in the maintenance stage. Moreover, the former has a longer service life, so the induction-healed pavement has a more sustainable development prospect from the perspective of energy consumption analysis.

### 4.2. Greenhouse Gas Emission Impact Assessment

**Figure 9:**
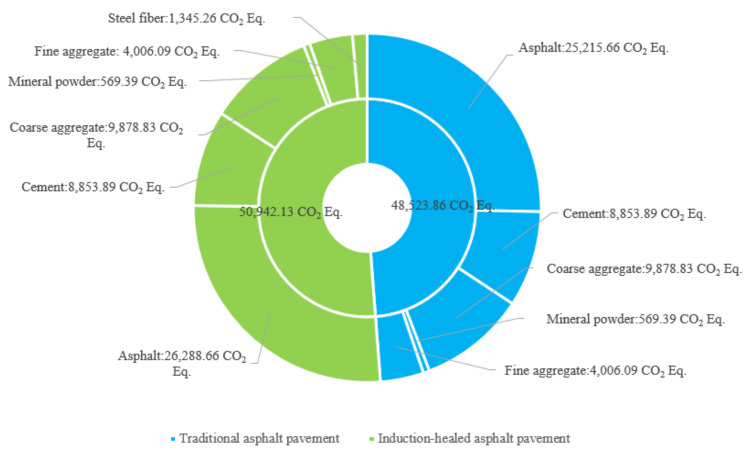
Greenhouse gas emissions of two asphalt pavements in the material manufacturing stage.

**Figure 10:**
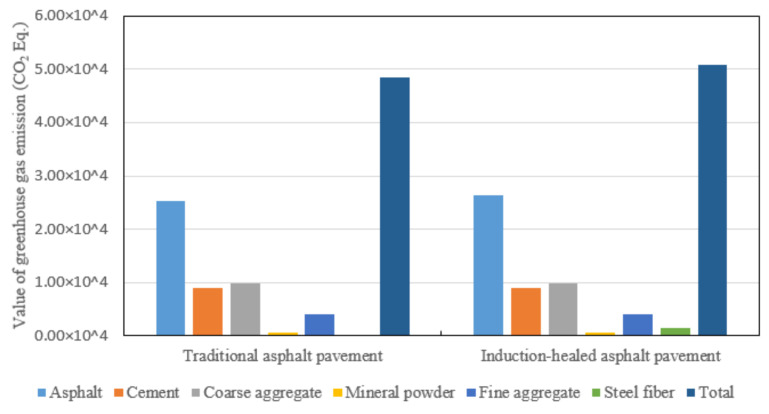
Fluctuation chart of greenhouse gas emissions of two asphalt pavements in the material manufacturing stage.

In the material manufacturing stage of raw materials, the GHG emissions comparison between induction-healed pavement and traditional asphalt pavement is shown in Figure 9 and Figure 10. The pie chart shows the proportion of GHG emissions in manufacturing and transportation for each material, and the bar chart shows the difference in the manufacturing and transportation GHG emissions for each material. Among all raw materials, asphalt is the largest source of GHG emissions. Meanwhile, the total emissions of the two asphalt pavements in this stage are similar, and the equivalent CO_2_ emissions sequence is: asphalt, cement, coarse aggregate, fine aggregate, steel fiber, mineral powder.

**Figure 11:**
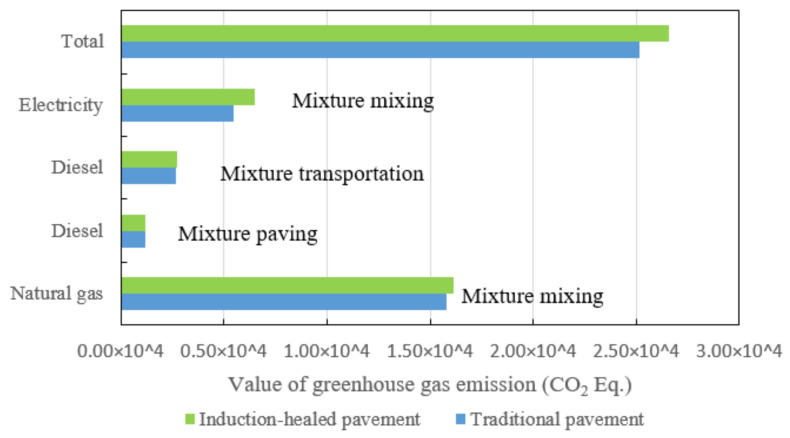
Greenhouse gas emissions comparison of two asphalt pavements’ paving stages.

In the road paving stage, GHG emissions of the two asphalt pavements the are shown in Figure 11. Comparing the GHG emissions of the two types of asphalt pavement, natural gas produces the most GHG emissions, followed by electricity and diesel. The total emissions from induction-healed pavement are slightly higher than those from traditional pavement due to the addition of steel fiber to improve the quality and density of the mixture, as well as the adjustment of the mixing process.

**Figure 12:**
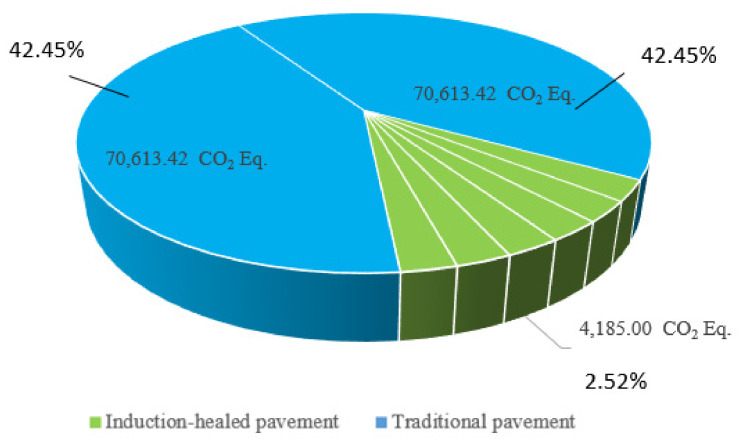
Greenhouse gas emission of two overhauls of traditional pavement and six electromagnetic induction maintenance sessions of induction-healed pavement.

Similar to the energy consumption in the maintenance stage, the equivalent CO_2_ emissions of the traditional pavement is still higher than that of the induction-healed pavement. As shown in Figure 12, GHG emissions from the former are 5.6 times that of the latter. During the service life of traditional pavement, the emissions generated by maintenance measures such as pre-maintenance, minor repair and medium repair of the maintenance stage are not taken into account, and the actual emissions generated by the former are much higher than those of the latter.

**Figure 13:**
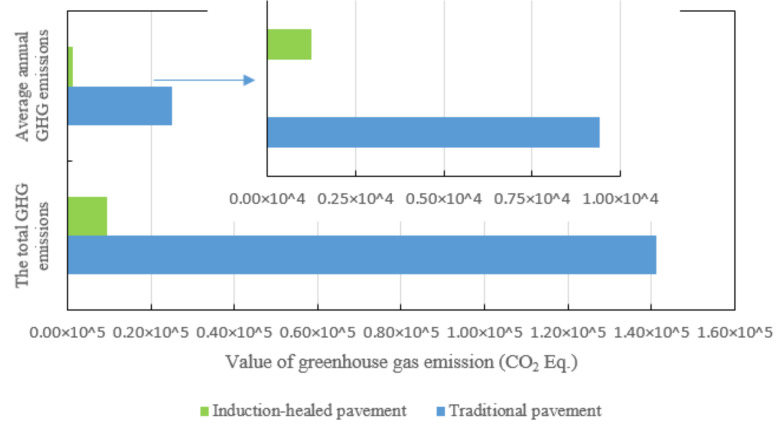
Greenhouse gas emissions of two asphalt pavements’ maintenance in their design life.

Figure 13 shows the total GHG emissions and the average annual GHG emissions of two asphalt pavement during their service life.

The average annual emissions from traditional pavement are 7.5 times those from induction-healed pavement. From the point of view of the maintenance stage, the environmental load of induction-healed pavement is much lower than that of traditional asphalt pavement. Moreover, under the same conditions, the former can realize the longer service life of asphalt pavement more quickly and conveniently. Therefore, induction-healed pavement should be a sustainable pavement technology.

**Figure 14:**
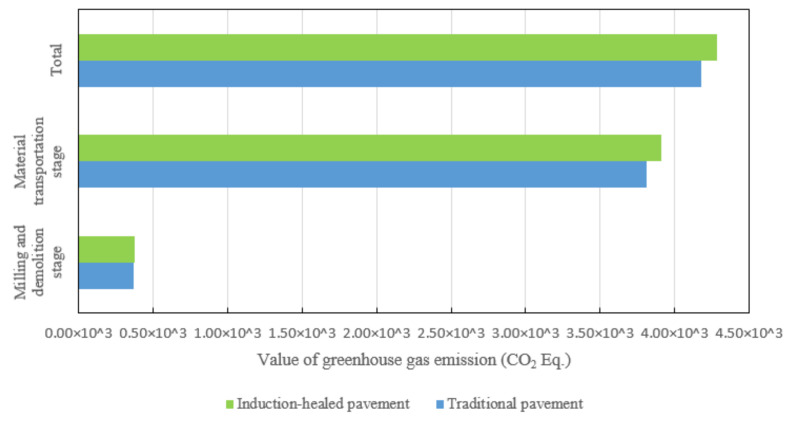
Greenhouse gas emissions of two asphalt pavements’ in milling and demolition stages.

Figure 14 shows the GHG emissions comparison between the two asphalt pavements’ milling and demolition stages. The GHG emissions from induction-healed pavement are slightly higher than those from traditional pavement due to the addition of steel fiber increasing the quality and density of mixture and consuming more fossil energy, thus increasing GHG emissions.

**Figure 15:**
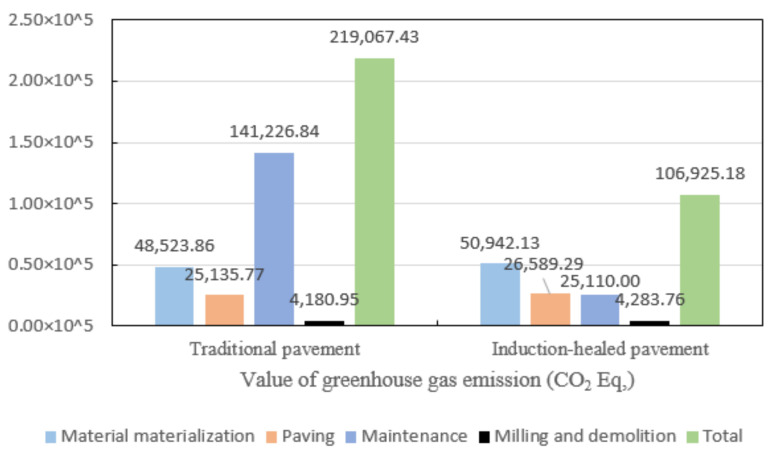
Greenhouse gas emissions of two asphalt pavements in their whole life cycle.

Based on the GHG emissions of the four stages, the GHG emissions of the pavement life cycle is shown in Figure 15. It can be seen from the figure that the GHG emissions from traditional pavement are about two times those of induction-healed pavement. This is mainly because, in the maintenance stage, the GHG emissions generated by two overhauls of traditional asphalt pavement are much higher than those generated by electromagnetic induction maintenance vehicles.

## 5. Conclusions

In this study, two different cases were used to compare induced healing with conventional milling and lay-up techniques to assess the sustainable development prospects of induced healing. The LCA method is used to divide the pavement life cycle into four stages: the material manufacturing stage, paving stage, maintenance stage, milling and demolition stage. The energy consumption and the GHG emissions in each stage were analyzed, respectively, and the following conclusions were drawn:

1. Among the two kinds of asphalt pavements’ service life, the energy consumption in raw material manufacturing stage of induction-healed asphalt pavement is the highest, while that of the maintenance stage of traditional asphalt pavement is the highest. Combined with the energy consumption of the four stages, the total energy consumption of the latter is about 2.5 times that of the former.

2. In the service life of the two kinds of asphalt pavement, the GHG emissions in the raw material manufacturing stage of induction-healed asphalt pavement is the highest, while that of the maintenance stage of traditional asphalt pavement is the highest. Combined with the GHG emissions of the four stages, the total GHG emissions of the latter are about two times those of the former.

3. In the maintenance stage, the single maintenance energy consumption of induction-healed pavement accounts for 1.4% of the single overhaul of traditional asphalt pavement. The energy consumption generated by the six EMI maintenance sessions of the former accounts for 4.3% of the two overhauls of the latter in its whole service life cycle.

4. In the maintenance stage, the GHG emissions from single maintenance of induction-healed pavement account for 5.9% of the single overhaul of traditional asphalt pavement. The GHG emissions generated by the six EMI maintenance sessions of the former account for 17.8% of the two overhauls in whole the service life cycle of the latter.

5. In the service life of the two kinds of asphalt pavement, the induction-healed pavement is more energy efficient and environmentally friendly than the traditional asphalt pavement. Moreover, under the same conditions, the induction healing technology can achieve longer service life of asphalt pavement faster and more easily. Therefore, induced healing pavement should be a sustainable pavement technology.

6. The methodology used for this study (converted to standard coal and using the LCA model) is likely to be implemented in future studies to obtain environmental emissions from different pavement maintenance methods.

## Figures and Tables

**Table 1:** Energy inventory involved in road construction.

Energy Types	Coefficient	Heat Energy	CO_2_ Emission Factor
Standard coal	1.000	29.270 MJ/kg	2.782 kg/kg
Heavy oil	1.429	41.816 MJ/kg	3.247 kg/kg
Gasoline	1.471	43.070 MJ/kg	2.996 kg/kg
Diesel	1.457	42.652 MJ/kg	3.171 kg/kg
Natural gas	1.330	38.929 MJ/kg	2.695 kg/kg
Electricity	0.123 kg/(kw·h)	3.596 MJ/(kw·h)	0.972 kg/(kw·h)

**Table 2:** Production mix ratio of ordinary asphalt concrete pavement.

Type	Aggregate		Mineral Powder	Cement	Asphalt
	3.5–22 mm	0–3.5 mm			Asphalt–Aggregate Ratio
GAC-16C	71	25	2	2	4.7/4.9

**Table 3:** List of raw material production.

Raw Material		Energy Consumption	CO_2_ Emissions
		MJ/t	Kg/t
Styrene-butadiene-styrene (SBS) modified asphalt	Matrix asphalt	4736.6	273.1
	SBS modifier	4286.5	55.0
	Modification processing	961.2	131.1
	Total	9984.3	459.2
Portland cement		3980.72	377.06
Coarse aggregate		108.59	9.02
Mineral powder		329.57	24.50
Fine aggregate		145.18	13.79
Steel fiber		423.41	50.33

**Table 4:** Transportation list of raw materials.

Raw Material	Distance	Single Load	Fuel Consumptionsmakal L/100 km (Diesel)		Energy Consumption	CO_2_ Emissions
	km	t	Full Load	No Load	MJ/t	Kg/t
Asphalt	67.3	50	38	30	33.573	2.496
Aggregate	115.4	100	39	29	28.784	2.140
Cement	210.7	100	39	29	52.555	3.907
Steel fiber	84.5	100	39	29	21.077	1.567

**Table 5:** Energy consumption in the preparation of asphalt mixture.

Energy	Traditional Pavement		Induction-Healed Pavement	
	MJ/t	1220 t/km	MJ/t	1250 t/km
Natural gas (kg)	4.802	5858.44	4.793	5991.25
Electricity (kw·h)	4.619	5635.18	5.356	6695.00

**Table 6:** Machinery list of paving stage.

	Model	Number of Devices	Workload/daysmakal (km)	Fuel Consumption/day (L, Diesel Fuel)	Fuel Consumption/km (kg, Diesel Fuel)
Paver	Vogler 2100-2 L	1	1.1	200	152.73
Double steel roller	Dynapac CC6200	1	2	250	105
Single steel roller	CLG 620H	2	2	48.25	40.53
Rubber roller	XP smakal303 K	1	2.5	220	73.92

**Table 7:** Maintenance schedule.

Year	Induction-Healed Pavement	Year	Traditional Pavement
0	Initial construction	0	Initial construction
5	Induction healing		
8	Induction healing	7	Mill and overlay
11	Induction healing		
13	Induction healing	12	Mill and overlay
15	Induction healing		
17	Induction healing		

**Table 8:** Energy consumption and greenhouse gas emission inventory in the structural demolition stage.

	Distance	Single Load	Fuel Consumption L/100 km (Diesel)		Energy Consumption	CO_2_ Emissions
	km	t	Full load	No load	MJ/t	Kg/t
Dump Truck	40	15	25	18	42.06	3.13
Milling Planer			0.11 L/t		4.03	0.30

## Data Availability

Not applicable.
